# Aging and Age-Related Disorders: From Molecular Mechanisms to Therapies

**DOI:** 10.3390/ijms20133280

**Published:** 2019-07-03

**Authors:** Vladimir I. Titorenko

**Affiliations:** Department of Biology, Concordia University, Montreal, 7141 Sherbrooke Street, West, QC H4B 1R6, Canada; vladimir.titorenko@concordia.ca; Tel.: +1-514-848-2424

Our understanding of the molecular mechanisms underlying cellular and organismal aging and aging-associated pathology has advanced greatly in recent years [1,2,3,4,5]. A body of evidence supports the notion that these mechanisms have been conserved during evolution [6,7,8,9,10]. The significant advances in knowledge about evolutionarily conserved mechanisms of aging and aging-associated disorders have paved the way to the establishment of quantitative biomarker signatures of human aging [11,12,13,14,15]. Validated biomarker signatures of aging could be used to develop dietary, nutraceutical, and pharmacological interventions as therapies for slowing the aging process, preventing disorders of old age, and extending the healthy lifespan in humans [16,17,18,19,20]. This *International Journal of Molecular Sciences* Special Issue was designed to capture the current state of knowledge and to discuss the recent progress in the understanding of molecular and cellular mechanisms, diagnostics, and therapies of aging and disorders of aging. Seven original research and review articles of the Issue provide important insights into the strategies used by evolutionarily diverse organisms for coordinating various longevity-defining cellular and organismal processes, and critically evaluate mechanisms through which pharmacological aging-delaying interventions regulate such coordination. These articles outline the most important unanswered questions and directions for future research in the vibrant and rapidly-evolving fields of mechanisms of biological aging, aging-associated disorders and aging-delaying therapies.

Using ribose-induced glycated reconstructed human skin, Balansin Rigon et al. demonstrate that the accumulation of advanced glycated end-products (AGEs) in the skin coincides with significant changes in the ultrastructural patterns of the *stratum papillare* dermal compartment and all epidermal compartments of the skin, which include *stratum reticulare*, *stratum corneum*, *stratum granulosum*, *stratum spinosum,* and *stratum basale* [21]. However, the build-up of AGEs in the reconstructed human skin has no effect on the shape and number of fibroblasts within each of these dermal and epidermal compartments. They also show that the concentrations of a number of adhesion and skin barrier protein components have been significantly altered in the reconstructed human skin that accumulates AGEs. The authors report that the relative abundance of several lipid classes is changed and the abundance of unsaturated trans fatty acids is increased in the *stratum corneum* epidermal compartment of this AGE-rich skin. Yet, the AGE-rich reconstructed human skin does not exhibit significant changes in the skin barrier function. Based on these findings, Balansin Rigon et al. concluded that ribose-induced glycated reconstructed human skin is a valuable nonclinical model of skin aging and age-related skin diseases. This model can be used to get insights into how the glycation of the extracellular matrix within the human skin can affect the skin’s morphological pattern, molecular composition, and barrier function. This model can also be implemented to develop novel therapies for age-related human skin diseases, such as diabetes, psoriasis, and skin cancer.

Melatonin is a hormone whose secretion declines with age. The essential roles of melatonin in the regulation of human circadian rhythms and sleep, and in the adaptive cellular response to oxidative molecular damage are well known. Hardeland provides deep insights into mechanisms through which melatonin, a known immunostimulatory agent, regulates the pro-inflammatory and anti-inflammatory networks in mammals and humans [22]. Melatonin orchestrates the pro-inflammatory networks by promoting the release of several pro-inflammatory cytokines. Under certain physiological conditions; however, melatonin can suppress inflammation by hindering nitric oxide release, inhibiting cyclooxygenase-2 involved in the conversion of arachidonic acid to prostanoids and prostaglandins, impeding NLRP3 inflammasome and gasdermin D to decelerate an inflammatory mode of cell death, turning off the toll-like receptor-4 signaling pathway to weaken a pro-inflammatory response to infectious or non-infectious stimuli, lowering the intensity of mTOR (the mechanistic or mammalian target of rapamycin protein complex) signaling implicated in the regulation of innate immune homeostasis, impeding inflammatory cytokine secretion via senescence-associated secretory phenotype machinery, and promoting anti-amyloidogenic responses. The author points out that under certain physiological scenarios, melatonin can stimulate the anti-inflammatory network by activating the NAD-dependent protein deacetylase sirtuin-1, promoting the nuclear factor erythroid 2-related factor 2 pathway, suppressing the nuclear factor kappa-light-chain-enhancer of activated B cells pathway, accelerating secretion of some anti-inflammatory cytokines, and stimulating a transition of macrophages and microglia to the anti-inflammatory phenotype. The author emphasizes that many of these effects of melatonin on the pro-inflammatory and anti-inflammatory networks are linked to its ability to upregulate or downregulate a distinct set of microRNAs.

The hematopoietic stem cells (HSCs) in the bone marrow are progenitors of the red blood cells that are involved in oxygen transport and blood thrombocytes that are needed for blood clotting at the damaged site of a blood vessel. HSCs also serve as precursors of different cell types of the immune system and are, therefore, indispensable for the maintenance of the immune system. A body of evidence indicates that the ability of HSCs to self-renew and differentiate into the different cell types of the immune system declines with age and causes the development of various immune diseases. These aging-associated immune diseases include myeloid and lymphoid leukemias, anemia, adaptive immunity deterioration, autoimmunity, weakened resistance to infectious diseases, and primary vaccine failures. Lee et al. review various cellular events characteristic of HSC aging, reasons and underlying mechanisms for the development of these hallmark events of HSC aging, and different strategies that can be used for the rejuvenation of aged HSCs through the restoration of their dysregulated functionality [23]. The authors also outline two recently developed technologies used for studying HSCs, namely single-cell RNA-sequencing and single-cell transplantation.

Aging is associated with specific changes in the functionality of the blood-brain and blood-cerebrospinal fluid barriers (BBB and BCSFB, respectively), both of which mediate neuroimmune communications. Erickson and Banks explore recently discovered mechanisms underlying these aging-associated changes in BBB and BCSFB functions [24]. They also outline how aging influences the adaptive and innate immune systems (and related physiological processes) outside of the central nervous system. The authors discuss a network of interactions that exist between the BBB and the immune system, examine how these interactions are affected during aging, and analyze how the aging-associated changes in these interactions contribute to the development of neurological and neurodegenerative diseases of old age. They underscore that the knowledge about mechanisms through which the functionality of the BBB and its interactions with the immune system are altered during aging is essential for the understanding of the impact of these alterations on aging-associated cognitive decline and neurodegeneration. This knowledge is also instrumental in the development of novel therapies for neurological and neurodegenerative diseases of old age.

Fluvastatin is a synthetic antilipemic agent that inhibits hydroxymethylglutaryl-coenzyme A reductase; it has been approved for medical use as a drug for lowering plasma cholesterol concentrations and preventing cardiovascular disease. Valsartan is a synthetic antagonist of angiotensin type I receptors; it is used as a drug for the treatment of high blood pressure, heart failure, and diabetic kidney disease. Short-term treatment of middle-aged males with low doses of fluvastatin and valsartan is known to a cause a significant and long-lasting improvement of the arterial wall features that are characteristic of arterial aging. The fluvastatin- and valsartan-dependent effect on the arterial wall has been shown to coincide with increased telomerase expression, reduced inflammation, and lowered oxidative stress in the fluvastatin- and valsartan-treated individuals. These effects of fluvastatin and valsartan on telomerase, inflammation, and oxidative stress suggest that they may slow aging and aging-associated diseases in humans. Since aging-associated diseases are known to be linked to altered expression of a distinct subset of aging-related genes that are often called longevity genes, Janić et al. used quantitative real-time PCR of cells recovered from whole blood samples to investigate how a short-term treatment of middle-aged males with low doses of fluvastatin and valsartan (which were used separately or in combination) influences the expression of these genes [25]. They show that treatment with fluvastatin or valsartan significantly upregulates the expression of SIRT1, a NAD-dependent protein deacetylase that plays essential roles in aging and aging-associated diseases. They also report that a mixture of fluvastatin and valsartan significantly stimulates the expression of SIRT1, as well as the expression of PRKAA (a catalytic alpha subunit of the 5’-AMP-activated protein kinase) and KLOTHO (a β-glucuronidase that hydrolyzes steroid β-glucuronides). Based on these findings, the authors predict that a pairwise combination of low doses of fluvastatin and valsartan can be used to delay the onset of aging-associated disorders (or prevent such disorders) not only by lowering plasma cholesterol concentrations and preventing cardiovascular disease, maintaining low blood pressure and improving heart and kidney functions, but also by promoting the expression of the longevity genes encoding SIRT1, PRKAA, and KLOTHO proteins.

Mohammad et al. bring together an extensive body of experimental data to discuss the characteristic metabolic, signal transduction, gene expression, epigenetic, stress survival, and cell cycle regulation traits of quiescent adult stem cells in mammals and humans [26]. They explore how each of these traits can contribute to the quiescence, self-renewal, proliferation, and functionality of these cells. The authors analyze and critically evaluate an intricate network of cell-intrinsic mechanisms that control the establishment and preservation of these traits. They outline how this network of cell-intrinsic mechanisms regulates quiescence entry, maintenance, and exit in response to certain extrinsic pro-mitogenic and anti-mitogenic cues from the microenvironment of adult stem cells. The authors accentuate that, because the impairment of a balance between the quiescence, proliferation, and differentiation of adult stem cells underlies the pathophysiology of many aging-associated diseases, it is critically important to understand how this balance can be controlled to decelerate cellular and organismal aging and to delay the onset of diseases of old age.

Glycogen synthase kinase 3 (GSK3) is an evolutionarily conserved serine-threonine protein kinase that contributes to the regulation of cell metabolism, gene expression, differentiation, migration, apoptotic death, and survival because it phosphorylates many proteins involved in these cellular processes. GSK3 of the fruit fly *Drosophila melanogaster (D. melanogaster)*, which is encoded by the *shaggy* (*sgg*) gene, is known to be an essential contributor to the development and function of the nervous system. Differential transcription of the *sgg* gene in *D. melanogaster* yields at least 17 transcripts that encode no less than 10 different protein isoforms. Trostnikov et al. examine how a tissue-specific overexpression of the alternative *sgg* transcripts known to be expressed in the nervous system influences organismal longevity [27]. The alternative *sgg* transcripts *sgg-RA*, *sgg-RB*, *sgg-RG,* and *sgg-RO* were overexpressed in embryos, the fat body, muscles, and the nervous system. They provide evidence that the overexpression of *sgg-RA*, *sgg-RG,* and *sgg-RO* in each of these tissues has only a minor effect on the organismal lifespan, with the exception of *sgg-RA* overexpression in muscles and *sgg-RO* overexpression in embryos. In contrast, the overexpression of *sgg-RB* in embryos and the fat body is lethal, whereas *sgg-RB* overexpression in muscles and the nervous system shortens lifespans of both male and female flies. They demonstrate that *sgg-RB* overexpression in dopaminergic neurons has a strong accelerating effect on organismal aging, while *sgg-RB* overexpression in GABAergic neurons only marginally accelerates organismal aging. They also show that the observed abilities of a lifelong pan-neuronal *sgg-RB* overexpression to shorten organismal lifespan and accelerate aging of the nervous system coincide with its abilities to alter various aspects of mitochondrial structure and function, damage cytoskeleton structure, impair the integrity of neuronal bodies, lower neuronal synaptic activity, and decrease the nervous system-dependent locomotion of flies. Because the pan-neuronal *sgg-RB* overexpression causes premature aging of the nervous system already in young flies, the authors conclude that GSK3 plays the key role in linking the development, function, and aging of the nervous system to organismal longevity in *D. melanogaster*.
